# A Mobile Phone and Web-Based Intervention for Improving Mental Well-Being in Young People With Type 1 Diabetes: Design of a Randomized Controlled Trial

**DOI:** 10.2196/resprot.4032

**Published:** 2015-05-05

**Authors:** Janine Clarke, Veronica Vatiliotis, Charles F Verge, Jane Holmes-Walker, Lesley V Campbell, Kay Wilhelm, Judy Proudfoot

**Affiliations:** ^1^Black Dog Institute and UNSWSydneyAustralia; ^2^EndocrinologySydney Children's Hospital RandwickSydneyAustralia; ^3^School of Women’s and Children’s HealthUNSWSydneyAustralia; ^4^Westmead HospitalSydneyAustralia; ^5^The University of SydneySydneyAustralia; ^6^Diabetes & Metabolism DivisionGarvan Institute of Medical ResearchSydneyAustralia; ^7^Diabetes ServicesSt Vincent's HospitalSydneyAustralia; ^8^School of PsychiatryUNSWSydneyAustralia; ^9^Black Dog InstituteSydneyAustralia; ^10^Faces in the StreetUrban Mental Health and Wellbeing Research InstituteSt Vincent’s HospitalSydneyAustralia

**Keywords:** type 1 diabetes, depression, diabetes-related distress, Internet intervention, randomized controlled trial

## Abstract

**Background:**

Young people with type 1 diabetes experience elevated levels of emotional distress that impact negatively on their diabetes self-care, quality of life, and disease-related morbidity and mortality. While the need is great and clinically significant, a range of structural (eg, service availability), psychological (eg, perceived stigma), and practical (eg, time and lifestyle) barriers mean that a majority of young people do not access the support they need to manage the emotional and behavioral challenges of type 1 diabetes.

**Objective:**

The aim of this study is to examine the effectiveness of a fully-automated cognitive behavior therapy-based mobile phone and Web-based psychotherapeutic intervention (*myCompass*) for reducing mental health symptoms and diabetes-related distress, and improving positive well-being in this vulnerable patient group.

**Methods:**

A two-arm randomized controlled trial will be conducted. Young people with type 1 diabetes and at least mild psychological distress will be recruited via outpatient diabetes centers at three tertiary hospitals in Sydney, Australia, and referred for screening to a study-specific website. Data will be collected entirely online. Participants randomized to the intervention group will use the *myCompass* intervention for 7 weeks, while at the same time a control group will use an active placebo program matched to the intervention on duration, mode of delivery, and interactivity.

**Results:**

The primary outcome will be mental well-being (ie, depression, anxiety, diabetes-related distress, and positive well-being), for which data will be collected at baseline, post-intervention, and after 3 months follow-up. Secondary outcomes will be functional (work and social functioning and diabetes self-care), biochemical measures (HbA1c), and mental health self-efficacy. We aim to recruit 280 people into the study that will be conducted entirely online. Group differences will be analyzed on an intention-to-treat basis using mixed models repeated measures.

**Conclusions:**

We hypothesize that scores on the outcome measures will improve significantly for young people who use the mobile phone and Web-based intervention compared to the control group. *myCompass* is a public health intervention that is broadly available and free to use. If effective, the program has the capacity to provide convenient and accessible evidenced-based care to the large group of young people with type 1 diabetes who do not currently access the psychosocial support they need.

**Trial registration:**

Australian New Zealand Clinical Trials Registry: ACTRN12614000974606; https://www.anzctr.org.au/Trial/Registration/TrialReview.aspx?id=366607 (Archived by WebCite at http://www.webcitation.org/6YGdeT0Dk).

## Introduction

### Background

Type 1 diabetes is one of the most common chronic diseases of childhood [1]. It is a leading cause of global disease burden [2], and prevalence rates are increasing [3,4]. Insulin deficiency, the hallmark feature of type 1 diabetes, requires life-long management of a complex and demanding self-care regimen that aims to optimize glycemic control and prevent the onset of potentially fatal short- (eg, hypoglycemia and diabetic ketoacidosis) and long-term (eg, micro-vascular and macro-vascular disorders, peripheral nerve disease, and blood vessel disease) health complications [1]. How well young people cope with the challenges of type 1 diabetes, while simultaneously negotiating the normal developmental tasks of childhood, adolescence, and the transition to adulthood, has important implications for their overall physical health, psychological well-being, quality of life, and life expectancy [5].

People with type 1 diabetes feel challenged emotionally by the daily hassles, frustrations, and worries that stem from having a serious and chronic medical condition [6], and are at greater risk of common mental health problems, especially depression, than their peers without diabetes [7,8]. This is clinically important because of the links between psychological morbidity and such adverse health outcomes as greater diabetes symptom burden and functional impairment, decreased diabetes self-care, poorer glycemic control, higher rates of diabetes complications, and failure to transition from pediatric to adult diabetes services [9-11]. Even mild (subclinical) emotional distress has been demonstrated in longitudinal studies to predict “worse-than-expected” clinical and psychological outcomes in adulthood for young people with diabetes, including recurrent hospital admissions for diabetic ketoacidosis [12].

A meta-analysis of interventions for clinical and subclinical depression clearly demonstrated the benefits for mental health and diabetes outcomes [13], yet most young people with type 1 diabetes do not receive the psychological support they need to manage the emotional and behavioral challenges of their diabetes [14,15]. Barriers to obtaining psychological support include insufficient providers of mental health services, poor mental health literacy (ie, lack of knowledge of signs and symptoms), the prioritization of physical symptoms in traditional models of diabetes care, and a range of practical constraints (eg, time, lifestyle, and financial) [6]. Furthermore, there is evidence that many young people with type 1 diabetes find it difficult to discuss mental health issues with their health care providers [15], thus delaying and/or avoiding seeking psychosocial support. There is, therefore, considerable opportunity to improve mental and physical health outcomes for young people with type 1 diabetes by increasing access to psychosocial support that reduces geographic, temporal, and financial barriers to access, and offers advantages of user confidentiality and anonymity.

As an important part of the everyday lives of young people [16,17], the Internet has demonstrated its potential to overcome many of the barriers to accessing mental health services [18], evolving as a popular, efficient, and clinically effective means of delivering empirically supported psychological interventions to the public, including people with diabetes [19-22]. Young people report feeling empowered and comfortable exploring sensitive and stigmatized issues online [23], and online resources, including websites, forums, and social networking sites, are increasing in popularity as sources of mental health support [16,17,24]. Surprisingly, however, there is little research examining the efficacy of Internet delivered psychotherapeutic interventions for reducing distress and improving psychological well-being in young people with type 1 diabetes.

Therefore, the current study seeks to evaluate the feasibility, acceptability, and clinical effectiveness for improving mental well-being in young people with type 1 diabetes of a fully-automated mobile phone and Web-based intervention*, myCompass*. Grounded in cognitive behavior therapy (CBT), the *myCompass* program has been demonstrated in a randomized controlled trial (RCT) to reduce symptoms and functional impairment in members of the community with mild to moderate levels of depression, anxiety, and stress [25]. Additional pilot data from an uncontrolled study suggest that not only general distress but also diabetes-specific emotional problems are improved (unpublished data by Clarke, Proudfoot, and Ma, 2014).

### Study Aims and Hypotheses

Our primary hypothesis is that young people with type 1 diabetes who use the *myCompass* program for 7 weeks will report fewer mental health symptoms (depression, anxiety, and diabetes-related distress), and improved positive well-being compared to an active placebo control group. Our secondary hypothesis is that use of *myCompass* will lead to greater functional gains (diabetes self-care, and work and social functioning), and improvements in glycemic control than the comparison intervention.

## Methods

### Study Design

**Figure 1:**
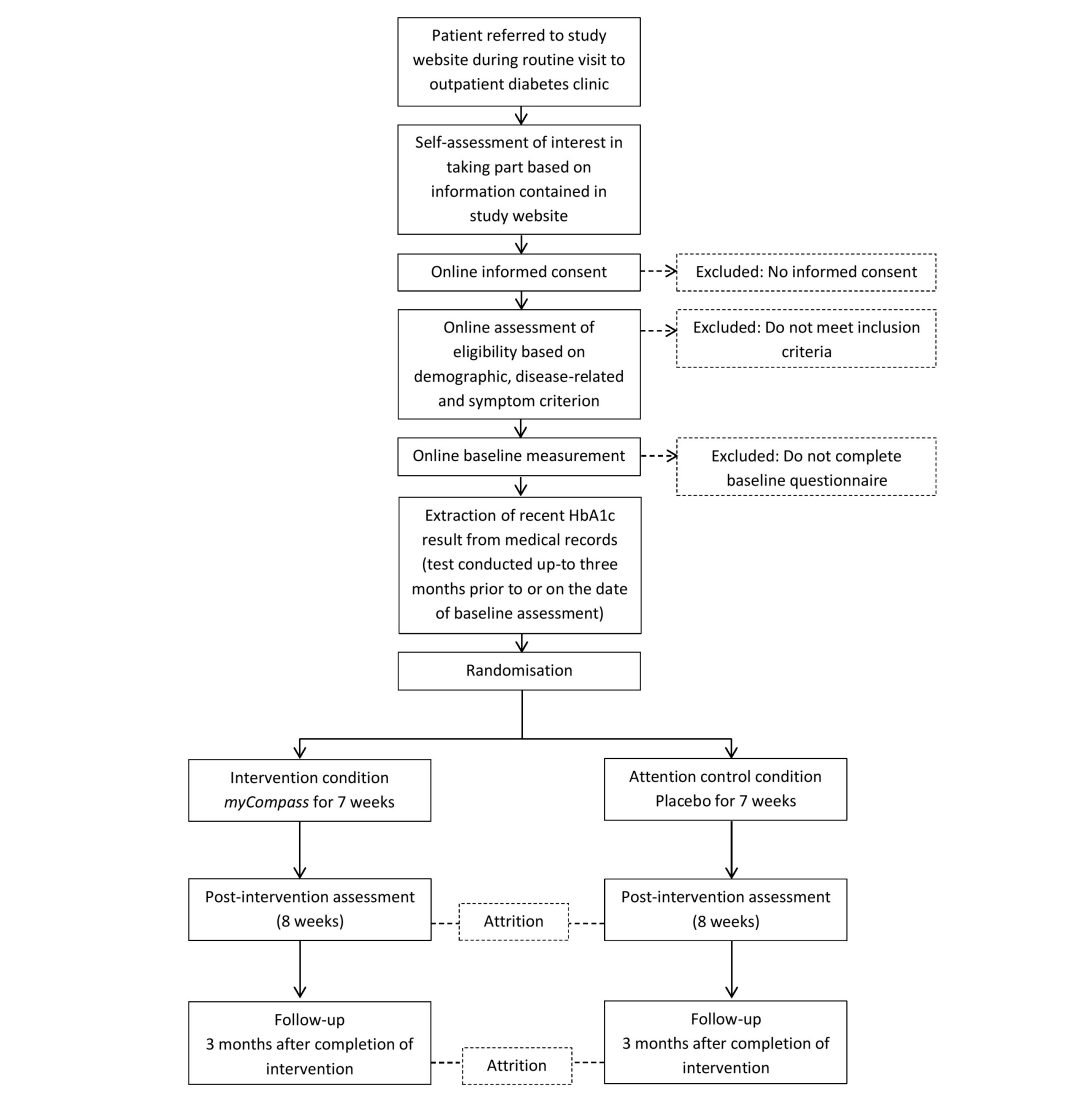
Study flow chart.

A 2 (conditions) x 3 (time) RCT design is planned. Participants will be randomized to either the *myCompass* intervention or an active placebo intervention, and each group will have full program access for 7 weeks. Outcomes will be assessed at baseline, post-intervention (8 weeks), and three months follow-up (Figure 1).

### Participants

#### Recruitment

Participants will be young people with type 1 diabetes recruited via diabetes services at three hospitals in Sydney, Australia: The Sydney Children’s Hospital, Westmead Hospital, and St Vincent’s Hospital. Clinical staff at each service will provide potential participants with written information about the study and an invitation to take part during a routine visit. Information will describe the study and instruct interested individuals to access a study-specific website to complete an online consent form and screening survey.

#### Eligibility

Young people will be eligible for the trial if they are an Australian resident aged between 16-25 years (inclusive), have an email address and Internet access (via mobile phone, and computer or tablet), were diagnosed with type 1 diabetes by a specialist clinician, and have at least mild symptoms of psychological distress. In light of research suggesting that mental health problems (especially those in the subclinical range) in people with diabetes may, in part, reflect disease-specific distress [26], and given calls for routine screening of both general and diabetes-specific distress in diabetes patients [27], a young person will meet our inclusion criteria if they have a mean score of ≥2 on the Diabetes Distress Scale (DDS) [28], and/or a total score of ≥5 on the on the Patient Health Questionnaire (PHQ) [29]. Exclusion criteria include the inability to read English easily, previous use of the *myCompass* intervention, and current psychotic symptoms (total score of ≥2 on the Psychosis Screening Questionnaire (PSQ) [30] (Textbox 1). Screening will be automatically stopped and appropriate feedback provided if any responses indicate ineligibility.

**Inclusion and exclusion criteria.**

Criteria

- Inclusion
  - Consent to participate
  - Age 16-25 years
  - Type 1 diabetes, diagnosed by an endocrinologist
  - Access to an Internet-enabled mobile phone and computer/tablet
  - Patient Health Questionnaire (PHQ-9) score of ≥5 and/or Diabetes Distress Scale score of ≥2
- Exclusion
  - Inability to read English with ease
  - Previous experience with the *myCompass* program
  - Psychotic symptoms
  - Non-residence in Australia

#### Ethical Concerns and Consent

The study protocol has been approved by the Ethics Committee at St Vincent’s Hospital, which is certified by the National Health and Medical Research Council in Australia (HREC/14/SVH/31), and research governance bodies at each of the participating hospitals. The protocol is registered with the Australia and New Zealand Clinical Trials Registry (ACTRN12614000974606).

### Intervention and Control

#### myCompass

**Figure 2:**
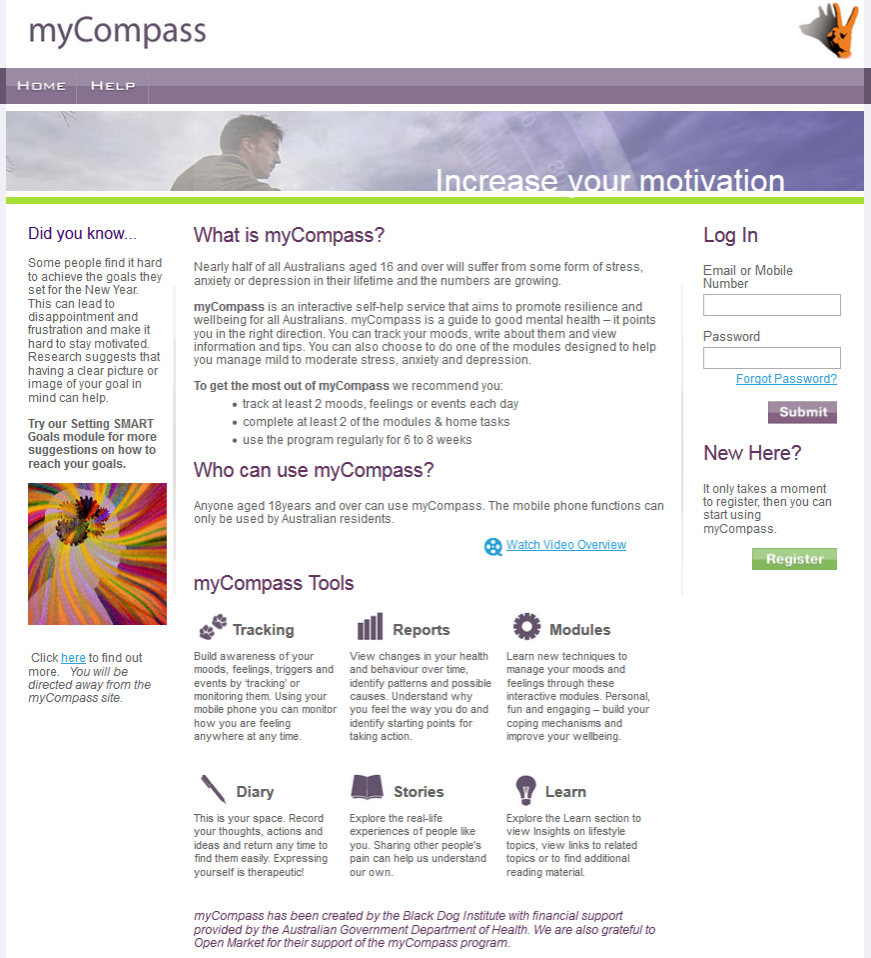
Screenshot of myCompass.

The *myCompass* program [31] is a fully-automated public health intervention with no therapist input that can be accessed via any Internet-enabled mobile phone, tablet, or computer (Figure 2). Developed by mental health researchers at the Black Dog Institute, the program assesses users’ self-reported mental health symptoms on registration, and delivers a personalized intervention that provides round-the-clock self-monitoring of moods and behaviors (via mobile phone, tablet, or computer), and access to twelve interactive evidence-based learning modules (via tablet and computer). The modules provide skills training drawn from cognitive behavioral, interpersonal, problem solving, and positive psychology therapies, and cover such topics as solving problems, managing fear and anxiety, tackling unhelpful thinking, dealing with stress and overload, and increasing pleasurable activities. Each module comprises three 10-minute sessions and has practice activities and home-tasks assigned. Users are encouraged to complete at least two modules, either of their own choosing or from recommendations provided on registration.

In addition, users can schedule short message service (SMS) or email reminders to facilitate self-monitoring. Reminders can be used to receive and print graphical feedback about self-monitoring (including contextual information) on mobile phones or computers to monitor change and assist identification of triggers. It is also possible to receive helpful facts, mental health-care tips, or motivational statements by SMS or email. Registering to use the program is free, and users are not billed for the SMSs they receive. A detailed description of the *myCompass* intervention is provided in Proudfoot et al [25].

#### Active Placebo

The comparator intervention will be an Internet-delivered program called *LiveWell* which delivers health information about a range of topics including skin care, mobile phone use, home environment, casual work, healthy food, and relationships. Developed by the research team to match *myCompass* on duration and mode of delivery, the program also contains practice activities, home tasks, and factual SMS messages (sent to participants once-weekly), and a symptom check at 4 weeks, to replicate the interactivity of *myCompass*, but has no therapeutic content.

### Procedure

Participants will have access to the full intervention on their mobile phones and computer devices for 7 weeks. Although participants will be encouraged to use the programs ad libitum during the intervention period, it will be recommended that they complete at least two program modules in their own time. Assessment will be conducted completely online. At each assessment time-point, participants will receive an email asking them to log into the study website to complete the outcome measures. The email sent at 3-months follow-up will also prompt participants to visit their diabetes specialist for assessment of glycosylated haemoglobin (HbA1c).

### Randomization

Randomization to either *myCompass* or the active placebo intervention will be carried out after baseline measurement, according to a sequence generated by a computerized random-number generator [32] using permutated blocks of 2, 4, and 8. The randomization process will be facilitated by a researcher not involved with the study. Participants will receive login details for their respective interventions by email.

### Measures

The assessments that will be completed at baseline, post-treatment, and 3-months follow-up are summarized in Table 1. Demographic data will include gender, age, marital status, highest education level, and whether the young person is currently working and/or studying. Disease-related information will include age at diagnosis, treatment modality, and diabetes complications status. Participants will also nominate their general practitioner (GP) and diabetes specialist in order to facilitate risk management and the collection of HbA1c results by research personnel.

**Table 1:** Measures used at screening, baseline, post intervention, and 3- month follow-up.

Measure	Screening	Baseline	Post-test	3 months
Demographic and disease-related variables		X^a^		
Psychosis Screening Questionnaire (PSQ)	X			
Patient Health Questionnaire-9 (PHQ-9)	X		X	X
Generalized Anxiety Disorder-7 (GAD-7)		X	X	X
Diabetes Distress Scale (DDS)	X		X	X
Warwick-Edinburgh Mental Well-being Scale (WEMWBS)		X	X	X
Work and Social Adjustment Scale (WSAS)		X	X	X
Summary of Diabetes Self-Care Activities (SDSCAS)		X	X	X
Hyperglycemia/Hypoglycemia scale		X	X	X
Glycosylated haemoglobin (HbA1c)		X	X	X
Mental Health Self-Efficacy Scale (MHSES)		X	X	X

^a^The X indicates the time-points at which the specific measure will be administered.

### Primary Outcome Measures

#### Depression Symptoms

The Patient Health Questionnaire-9 (PHQ-9) [29] contains 9 items assessing the Diagnostic and Statistical Manual of Mental Disorders (DSM)-IV criteria for major depressive disorder (MDD). The scale has excellent psychometric properties [33], and identifies similar rates of MDD when compared to semi-structured clinical interviews of DSM criteria in both adults [34] and adolescents [35]. It is used widely as a screening tool for depression, and is frequently included as outcome measures in studies of online interventions (eg, [36,37]). Scores of 5, 10, 15, and 20 are used as cut-off points for mild, moderate, moderately severe, and severe depressive symptoms, respectively.

#### Anxiety Symptoms

The Generalized Anxiety Disorder-7 Questionnaire (GAD-7) [38] contains 7 items assessing DSM-IV criteria for generalized anxiety disorder (GAD). The scale is well-validated as a screener for GAD [33], used frequently in an online format (eg, [36,37]), and shows good sensitivity and specificity for anxiety disorders generally [39]. Scores of 5, 10, and 15 represent cut-off points for mild, moderate, and severe anxiety symptoms, respectively.

#### Diabetes-Related Distress

The Diabetes Distress Scale (DDS) [28] is a 17-item scale that assesses the following four areas of diabetes-related emotional distress (1) emotional burden, (2) physician-related distress, (3) regimen-related distress, and (4) diabetes-related interpersonal distress. Scores on the DDS are calculated as the mean of all items and range from 1-6, with scores of >2 indicating “*little or no distress*”, and ≥3 indicating “*high distress*”. Data support the psychometric adequacy of the DDS when used in adult and adolescent samples [28,40].

#### Well-being

The Warwick-Edinburgh Mental Well-being Scale (WEMWBS) [41] is a 14-item scale that measures mental well-being through the concepts of positive affect, psychological functioning, and interpersonal relationships, and is validated for measuring mental well-being in young people aged ≥16 years [42]. Scores range from 14-70, with higher scores indicating more positive mental well-being.

### Secondary Outcome Measures

#### Work and Social Adjustment

The 5-item Work and Social Adjustment Scale (WSAS) [43] is a measure of the impact of mental health problems on daily functioning in the following five domains (1) work, (2) social leisure activities, (3) private leisure activities, (4) home management, and (5) personal relationships. Scores range from 0-40, with higher scores indicating poorer adjustment. Meyer et al [44] provide data supporting the psychometric adequacy of the WSAS when used in an online format.

#### Diabetes Self-Care

The 11-item Summary of Diabetes Self-Care Activities Scale (SDSCAS) [45] will be used to measure diabetes self-management. Participants rate how many days out of the past seven they have engaged in such activities as healthy eating, exercise, testing blood sugar, and foot care. Mean scores are calculated for each area and range between 0-7, with higher scores representing better self-care. Reviews support the reliability and validity of the SDSCAS as a self-report measure of diabetes self-management [45].

#### Glycemic Control

The 7-item Hyperglycemia Scale and 7-item Hypoglycemia Scale [46] will be used to assess participants’ self-reports of symptoms associated with high and low blood glucose, respectively. Additionally, as an objective indictor of glycemic control, participants’ HbA1c results will be retrieved from medical records (see Figure 1). The measurement of HbA1c provides an index of glycemic control over the preceding 2-3 months, and is useful for evaluating whether a person has achieved and maintained their treatment targets, as well as estimating their risk of chronic diabetes complications [47].

### Process Measures

#### Mental Health Self-Efficacy

The Mental Health Self-Efficacy Scale (MHSES) assesses people’s confidence in managing issues relating to their mental health using six, 10-point Likert scale items. Item scores are summed to obtain an overall measure (ranging from 6-60), with higher scores indicating greater mental health self-efficacy. The MHSES yields reliable and valid data, and is sensitive to change [48].

#### Program Usage

Usage will be examined for the *myCompass* group with respect to three indices, namely, frequency of logins, frequency of self-monitoring, and number of modules attempted.

### Risk Management Protocol

If any participant indicates a significant worsening of their psychological distress (defined as a score of >19 on the PHQ-9 either midway through the intervention period, at post-intervention, or follow-up) they will be sent an email from the research team advising them to contact their GP to arrange face-to-face support. A second email, sent 3 days later, will seek confirmation that contact with the GP has been made. If no contact has been made, or if no reply email is received, participants will be informed by email that the principal investigator of the study will contact their nominated GP to recommend they receive appropriate face-to-face support.

## Results

### Sample Size

The RCT of *myCompass* yielded an average between-group effect size on symptom outcomes of d=0.5 [25]. Van Bastellar et al [22] also reported controlled effect sizes of an online intervention for people with diabetes in the vicinity of d=0.5 for diabetes-related distress and depressive symptoms. Assuming an attrition rate of 50% [49,50], a total sample of 240 participants at follow-up (120 per arm) is the minimum required to detect between-group differences on self-report outcomes of .5 standard deviations with 80% power.

Nevertheless, we will aim for a final sample size 280 participants (140 per arm), as we have pilot data showing that this will enable us to detect a clinically meaningful decrease in HbA1c of 0.5% with power at 80%. This is based on analysis of clinic patients at Sydney Children’s Hospital, revealing a standard deviation of 1.04 for the change in HbA1c over 3 months. Recruitment is currently underway for this study.

### Statistical Analysis

Analyses will be completed with SPSS 22 software. Chi-squares (categorical variables) and *t* tests (continuous variables) will be used to compare demographic and disease-related variables and baseline scores on the outcome measures for the intervention and attention control groups. Similar analyses will be performed comparing participants who do (non-dropouts) and do not (dropouts) return completed questionnaires at each of the post-intervention and follow-up assessments to explore possible biases in study attrition.

Outcomes at each time point will be analyzed on an intention-to-treat basis using linear mixed modeling (LMM) [51], with time points as a within-group factor and intervention as a between-group factor. In LMM, incomplete cases are included in the analysis, and all available data is used to obtain parameter estimates. The interaction of time and study condition will be of primary interest in each analysis, with a significant interaction indicating a group difference in the pattern of change over time in the outcome of interest. Significant interactions will be explored using sets of Bonferroni adjusted comparisons of the two groups at post-intervention and 3-month follow-up. All effects will be tested at *P*<.05, with adjustment according to the number of contrasts in each set. Within- and between-group effect sizes will be calculated using Cohen’s d (based on the pooled standard deviation).

## Discussion

### Principal Findings

Reviews have highlighted the need for flexibility and innovation in reducing the substantial unmet need for psychosocial care in young people with type 1 diabetes [6,15]. To our knowledge, this study will be the first to examine the effectiveness of a fully-automated, self-help intervention that is generic in its content and delivered via the Internet to computers, tablets, and mobile phones for reducing mental health symptoms and improving mental well-being in this vulnerable patient group. *myCompass* is a public health intervention of demonstrated efficacy [25]. We hypothesize that general and diabetes-specific distress and psychological well-being will improve significantly in young people randomized to *myCompass* for 7 weeks compared to those randomized to an active placebo intervention.

Intervening to reduce psychological distress in young people with type 1 diabetes is important as emotional difficulties are associated with poorer self-care and high disease-related morbidity and mortality [9-11]. Furthermore, in the absence of treatment, psychological distress during the transition from childhood to adulthood may persist throughout life, thereby substantially increasing the longer-term personal and societal burden of the disease [12]. The preferred communication media of young people [52], delivering mental health care via the Internet and mobile phones may be particularly attractive to many young patients who are reluctant to access traditional face-to-face supports. Moreover, if found to be effective, *myCompass* is broadly available free of charge and could potentially reach large numbers of young people with type 1 diabetes for whom service availability and cost are major barriers to access.

Previous studies of psychological interventions for people with diabetes have tended to focus on depressive symptoms and diabetes-specific outcomes (eg, diabetes-related distress and diabetes self-care). By taking a broader approach and examining general and disease-specific distress (depression, anxiety, and diabetes-related distress), functional outcomes (work and social adjustment, and diabetes self-care), and positive mental health, this study provides a comprehensive evaluation of the effects of the web and mobile phone program on variables known to correlate with quality of life and health outcomes in people with diabetes [53,54]. An additional strength of this study is the examination of potential moderators (eg, demographic and disease-related variables, and mental health self-efficacy) of the effect of the intervention on outcome measures. These analyses may assist in identifying the young people with type 1 diabetes who are most likely to benefit from psychosocial support delivered via the Internet.

### Limitations

Recruitment of participants through tertiary hospital diabetes outpatient clinics is an obvious limitation of this study, and means that our findings may not generalize to young people with type 1 diabetes managed outside the hospital system. Nevertheless, because it can be difficult to recruit young people into RCTs [55], and given our target sample size of 280, we feel that the proposed recruitment strategy will ensure timely and cost-effective data collection. In addition, recruitment via hospitals will enable us to examine the potential advantages and disadvantages of including the *myCompass* intervention as part of routine specialist care for young people with type 1 diabetes.

Another possible limitation relates to the brevity of the study and the time intervals for measurement. Whereas the proposed assessments (immediately post-intervention and at 3-months follow-up) are sufficient to demonstrate change in the primary and secondary outcome measures, a longer-term follow-up is necessary to determine the consistency of the study findings and the pattern of the effects of the intervention over time. For this reason, consent will be sought from participants at 10 months for a supplementary 12 months follow-up assessment. Separate ethics approvals will be obtained for this part of the study and the data will be analyzed and reported independently of this project.

### Conclusions

The increasing prevalence of type 1 diabetes, the heightened risk of emotional difficulties in young people with the disease, and the substantial unmet need for psychosocial support, make a compelling case for trials of novel mental health interventions in this patient group. Using popular everyday tools such as mobile phones, tablets, and the Internet, this project will be the first to examine the effectiveness of a fully-automated, self-help intervention without diabetes-specific content for reducing distress and improving well-being for young people with type 1 diabetes. The *myCompass* intervention is widely accessible, with over 14,000 registrants since 2012. The program has the capacity to provide convenient, accessible (24 hours a day, 7 days a week), and clinically effective psychosocial care at no cost to young people who might otherwise have limited access to (or choose not to access) alternative sources of psychosocial support.

## Multimedia Appendix 1

CONSORT-EHEALTH checklist V1.6.2 [56].

## Abbreviations

CBT: Cognitive behavior therapy
DDS: Diabetes Distress Scale
DSM: Diagnostic and Statistical Manual of Mental Disorders
GAD: Generalized anxiety disorder
GP: General practitioner
HbA1c: Glycosylated haemoglobin
MDD: Major depressive disorder
MHSES: Mental Health Self-Efficacy Scale
PHQ: Patient Health Questionnaire
RCT: Randomized controlled trial
SDSCAS: Summary of Diabetes Self-Care Activities
SMS: Short message service
WEMWBS: Warwick-Edinburgh Mental Well-being Scale
WSAS: Work and Social Adjustment Scale
