# Supplementary material for: A Mobile Phone and Web-Based Intervention for Improving Mental Well-Being in Young People With Type 1 Diabetes: Design of a Randomized Controlled Trial
Source: JMIR Res Protoc. 2015 May 5;4(2):e50. doi: 10.2196/resprot.4032 (PMC4438197; doi:10.2196/resprot.4032)
Supplement: Supplementary file 1 [file resprot_v4i2e50_app1.pdf]

CONSORT-EHEALTH Checklist V1.6.2 Report

(based on CONSORT-EHEALTH V1.6), available at [http://tinyurl.com/consort-ehealth-v1-6].

4032

Date completed

11/14/2014 0:53:47

by

Janine Clarke

A mobile phone and web-based intervention for improving mental wellbeing in young people with type 1 diabetes: Design of a randomised controlled trial

TITLE

1a-i) Identify the mode of delivery in the title

"A mobile phone and web-based intervention"

1a-ii) Non-web-based components or important co-interventions in title

This item is NA as there are no non-web-based components or co-interventions.

1a-iii) Primary condition or target group in the title

"young people with type 1 diabetes"

ABSTRACT

1b-i) Key features/functionalities/components of the intervention and comparator in the METHODS section of the ABSTRACT

"Participants randomised to the intervention group will use the myCompass intervention for seven weeks, while at the same time a control group will use an active placebo program matched to the intervention on duration, mode of delivery and interactivity."

1b-ii) Level of human involvement in the METHODS section of the ABSTRACT

"Data will be collected entirely online."

1b-iii) Open vs. closed, web-based (self-assessment) vs. face-to-face assessments in the METHODS section of the ABSTRACT

"A two-arm randomised controlled trial will be conducted. Young people with T1D and at least mild psychological distress will be recruited via outpatient diabetes centres at three tertiary hospitals in Sydney, Australia, and referred for screening to a study-specific website. Data will be collected entirely online."

1b-iv) RESULTS section in abstract must contain use data

This paper outlines the protocol for a randomised controlled trial. The following information is provided. "The primary outcome will be mental well-being (i.e., depression, anxiety, diabetes-related distress and positive well-being), for which data will be collected at baseline, post-intervention and after 3 months follow-up. Secondary outcomes will be functional (work and social functioning and diabetes self-care) and biochemical measures (HbA1c) and mental health self-efficacy. We aim to recruit 280 people into the study that will be conducted entirely online. Group differences will be analysed on an intention-to-treat basis using mixed models repeated measures."

1b-v) CONCLUSIONS/DISCUSSION in abstract for negative trials

This item is not relevant as the paper outlines the protocol for a randomised controlled trial.

INTRODUCTION

2a-i) Problem and the type of system/solution

"most young people with T1D do not receive the psychological support they need to manage the emotional and behavioural challenges of their diabetes [14, 15]."

"There is, therefore, considerable opportunity to improve mental and physical health outcomes for young people with T1D by increasing access to psychosocial support that reduces geographic, temporal and financial barriers to access, and offers advantages of user confidentiality and anonymity."

"there is little research examining the efficacy of internet delivered psychotherapeutic interventions for reducing distress and improving psychological well-being in young people with T1D."

"Therefore, the current study seeks to evaluate the feasibility, acceptability and clinical effectiveness for improving mental well-being in young people with T1D of a fully-automated mobile phone and web-based intervention, myCompass."

2a-ii) Scientific background, rationale: What is known about the (type of) system

"Young people report feeling empowered and comfortable exploring sensitive and stigmatised issues online [23], and online resources, including websites, forums, and social networking sites, are increasing in popularity as sources of mental health support [16, 17, 24]."

"Grounded in cognitive behaviour therapy (CBT), the myCompass program has been demonstrated in a randomised controlled trial (RCT) to reduce symptoms and functional impairment in members of the community with mild-to-moderate levels of depression, anxiety and stress [25]."

"Young people report feeling empowered and comfortable exploring sensitive and stigmatised issues online [23], and online resources, including websites, forums, and social networking sites, are increasing in popularity as sources of mental health support [16, 17, 24]."

METHODS

3a) CONSORT: Description of trial design (such as parallel, factorial) including allocation ratio

"Our primary hypothesis is that young people with T1D who use the myCompass program for 7 weeks will report fewer mental health symptoms (depression, anxiety, and diabetes-related distress) and improved positive well-being compared to an active placebo control group. Our secondary hypothesis is that use of myCompass will lead to greater functional gains (diabetes self-care and work and social functioning) and improvements in glycaemic control than the comparison intervention."

3b) CONSORT: Important changes to methods after trial commencement (such as eligibility criteria), with reasons

This item is not relevant as the paper outlines the protocol for a randomised controlled trial.

3b-i) Bug fixes, Downtimes, Content Changes

The submitted manuscript describes the protocol for a randomised controlled trial. Hence, this item is not relevant at this stage.

4a) CONSORT: Eligibility criteria for participants

"Young people will be eligible for the trial if they are an Australian resident aged between 16 and 25 years (inclusive), have an email address and internet access (via mobile phone and computer or tablet), were diagnosed with T1D by a specialist clinician, and have at-least mild symptoms of psychological distress. In light of research suggesting that mental health problems (especially those in the subclinical range) in people with diabetes may in part reflect disease-specific distress [26], and given calls for routine screening of both general and diabetes-specific distress in diabetes patients [27], a young person will meet our inclusion criteria if they have a mean score > 2 on the Diabetes Distress Scale (DDS, [28]) and/or a total score > 5 on the on the Patient Health Questionnaire (PHQ, [29])."

4a-i) Computer / Internet literacy

"have an email address and internet access (via mobile phone and computer or tablet)"

4a-ii) Open vs. closed, web-based vs. face-to-face assessments:

"Clinical staff at each service will provide potential participants with written information about the study and an invitation to take part during a routine visit. Information will describe the study and instruct interested individuals to access a study-specific website to complete an online consent form and screening survey."

4a-iii) Information giving during recruitment

"Information will describe the study and instruct interested individuals to access a study-specific website to complete an online consent form and screening survey."

4b) CONSORT: Settings and locations where the data were collected

"Participants will be young people with T1D recruited via diabetes services at three hospitals in Sydney, Australia: The Sydney Children's Hospital, Westmead Hospital, and St Vincent's Hospital."

4b-i) Report if outcomes were (self-)assessed through online questionnaires

"Assessment will be conducted completely online. At each assessment time-point, participants will receive an email asking him/her to log into the study website to complete the outcome measures."

4b-ii) Report how institutional affiliations are displayed

The institutional details of participating hospitals through which participants will be recruited are displayed on the study materials.

5) CONSORT: Describe the interventions for each group with sufficient details to allow replication, including how and when they were actually administered

5-i) Mention names, credential, affiliations of the developers, sponsors, and owners

"The program was developed by mental health researchers at the Black Dog Institute"

5-ii) Describe the history/development process

Details of the intervention are provided in the manuscript text, and readers are referred elsewhere to a more detailed description of the program.
"the myCompass program has been demonstrated in a randomised controlled trial (RCT) to reduce symptoms and functional impairment in members of the community with mild-to-moderate levels of depression, anxiety and stress [25]."
"A detailed description of the myCompass intervention is provided in Proudfoot et al. [25]."

5-iii) Revisions and updating

"The myCompass program (https://www.myCompass.org.au/) is a fully-automated public health intervention with no therapist input that can be accessed via any internet-enabled mobile phone, tablet or computer (see Figure 2)."

5-iv) Quality assurance methods

The methodology described to collect the data was used successfully in a previous randomised controlled trial of the myCompass program.
25. Proudfoot J, Clarke J, Birch M-R, Whitton AE, Parker G, Manicavasagar V, Harrison V, Christensen H, Hadzi-Pavlovic D. Impact of a mobile phone and web program on symptom and functional outcomes for people with mild-to-moderate depression, anxiety and stress: a randomised controlled trial. BMC Psychiatry 2013;13:312.

5-v) Ensure replicability by publishing the source code, and/or providing screenshots/screen-capture video, and/or providing flowcharts of the algorithms used

"The myCompass program (https://www.myCompass.org.au/) is a fully-automated public health intervention with no therapist input that can be accessed via any internet-enabled mobile phone, tablet or computer (see Figure 2)."

5-vi) Digital preservation

"The myCompass program (https://www.myCompass.org.au/) is a fully-automated public health intervention with no therapist input that can be accessed via any internet-enabled mobile phone, tablet or computer (see Figure 2)."

5-vii) Access

"Participants will have access to the full intervention on their mobile phones and computer devices for seven weeks. Although participants will be encouraged to use the programs ad libitum during the intervention period, it will be recommended that they complete at least two program modules in their own time."

5-viii) Mode of delivery, features/functionalities/components of the intervention and comparator, and the theoretical framework

"myCompass
The myCompass program (https://www.myCompass.org.au/) is a fully-automated public health intervention with no therapist input that can be accessed via any internet-enabled mobile phone, tablet or computer (see Figure 2). The program was developed by mental health researchers at the Black Dog Institute and assesses users' self-reported mental health symptoms on registration, and provides a personalised intervention that facilitates round-the-clock self-monitoring of moods and behaviours (via mobile phone, tablet or computer) and provides twelve interactive evidence-based skill building modules (via tablet and computer). Each module comprises three 10-minute sessions and has practice tasks assigned.
In addition, users can schedule short message service (SMS) or email reminders to facilitate self-monitoring; receive and print graphical feedback about their self-monitoring, including contextual information, on their phone or computer (to monitor change and assist identification of triggers); and elect to receive helpful facts, mental health-care tips or motivational statements by SMS or email. Registering to use the program is free, and users are not billed for the SMSs they receive. A detailed description of the myCompass intervention is provided in Proudfoot et al. [25].
Active placebo
The comparator intervention will be an internet-delivered program called LiveWell which delivers health information about a range of topics including skin care, mobile phone use, home environment, casual work, healthy food and relationships. Developed by the research team to match myCompass on duration and mode of delivery, the program also contains practice activities, home tasks and factual SMS messages (sent to participants once-weekly) and a symptom check at four weeks, to replicate the interactivity of myCompass, but has no therapeutic content. "

5-ix) Describe use parameters

"Participants will have access to the full intervention on their mobile phones and computer devices for seven weeks. Although participants will be encouraged to use the programs ad libitum during the intervention period, it will be recommended that they complete at least two program modules in their own time."

5-x) Clarify the level of human involvement

Human involvement in the trial is minimal and limited to technical support and distribution of email prompts for questionnaire completion.
"Assessment will be conducted completely online. At each assessment time-point, participants will receive an email asking him/her to log into the study website to complete the outcome measures."

5-xi) Report any prompts/reminders used

"Assessment will be conducted completely online. At each assessment time-point, participants will receive an email asking him/her to log into the study website to complete the outcome measures."

5-xii) Describe any co-interventions (incl. training/support)

This item is not relevant as there are no co-interventions.

6a) CONSORT: Completely defined pre-specified primary and secondary outcome measures, including how and when they were assessed

"The assessments that will be completed at baseline, post-treatment and 3-months follow-up are summarised in Table 2."
"Primary outcome measures
Depression symptoms: The Patient Health Questionnaire-9 (PHQ-9 [29]) contains 9 items assessing the Diagnostic and Statistical Manual of Mental Disorders (DSM)-IV criteria for major depressive disorder (MDD). The scale has excellent psychometric properties [32], and identifies similar rates of MDD when compared to semi-structured clinical interviews of DSM criteria in both adults [33] and adolescents [34]. It is used widely as a screening tool for depression, and is frequently included as outcome measures in studies of online interventions [e.g., 35, 36]. Scores of 5, 10, 15 and 20 are used as cut-off points for mild, moderate, moderately severe, and severe depressive symptoms.
Anxiety symptoms: The Generalized Anxiety Disorder-7 Questionnaire (GAD-7 [37]) contains 7 items assessing DSM-IV criteria for generalized anxiety disorder (GAD). The scale is well validated as a screener for GAD [32], used frequently in an online format [e.g., 35, 36], and shows good sensitivity and specificity for anxiety disorders generally [38]. Scores of 5, 10 and 15 represent cut-points for mild, moderate and severe anxiety symptoms.
Diabetes-related distress: The Diabetes Distress Scale (DDS [28]) is a 17-item scale that assesses four areas of diabetes-related emotional distress, namely; emotional burden, physician-related distress, regimen-related distress, and diabetes-related interpersonal distress. Scores on the DDS are calculated as the mean of all items and range from 1 to 6, with scores less than 2 indicating "little or no distress", and 3 or higher indicating "high distress". Data supports the psychometric adequacy of the DDS when used in adult and adolescent samples [28, 39].
Wellbeing: The Warwick-Edinburgh Mental Well-being Scale (WEMWBS [40]) is a 14-item scale that measures mental wellbeing through the concepts of positive affect, psychological functioning, and interpersonal relationships, and is validated for measuring mental well-being in young people aged 16 years and over [41]. Scores range from 14 to 70, with higher scores indicating more positive mental wellbeing.
Secondary outcome measures
Work and social adjustment: The 5-item Work and Social Adjustment Scale (WSAS [42]) is a measure of the impact of mental health problems on daily functioning in five domains: work, social leisure activities, private leisure activities, home management, and personal relationships. Scores range from 0 to 40, with higher scores indicating poorer adjustment. Meyer et al. [43] provide data supporting the psychometric adequacy of the WSAS when used in an online format.
Diabetes self-care: The 11-item Summary of Diabetes Self-Care Activities scale (SDSCAS [44]) will be used to measure diabetes self-management. Participants rate how many days out of the past seven they have engaged in such activities as healthy eating, exercise, testing blood sugar, and foot care. Mean scores are calculated for each area and range between 0 and 7, with higher scores representing better self-care. Reviews support the reliability and validity of the SDSCAS as a self-report measure of diabetes self-management [44].
Glycaemic control: The 7-item Hyperglycaemia Scale and 7-item Hypoglycaemia Scale [45] will be used to assess participants' self-reports of symptoms associated with high and low blood glucose, respectively. Additionally, as an objective indictor of glycaemic control, participants' HbA1c results will be retrieved from medical records (See Figure 1). The measurement of HbA1c provides an index of glycemic control over the preceding 2 to 3 months, and is useful for evaluating whether a person has achieved and maintained their treatment targets, as well as estimating their risk of chronic diabetes complications [46].
Process measures
Mental Health Self-efficacy: The Mental Health Self-efficacy Scale (MHSES) assesses people's confidence in managing issues relating to their mental health using six, 10-point Likert scale items. Item scores are summed to obtain an overall measure (ranging from 6 to 60), with higher scores indicating greater mental health self-efficacy. The MHSES yields reliable and valid data, and is sensitive to change [47].
Program usage: Usage will be examined for the myCompass group with respect to three indices, namely, frequency of logins, frequency of self-monitoring, and number of modules attempted."

6a-i) Online questionnaires: describe if they were validated for online use and apply CHERRIES items to describe how the questionnaires were designed/deployed

Where possible, outcome measures were selected on the basis of their previous use in online studies. It is unclear from the literature, and given the novelty of the study, whether all scales have been used in an online format e.g. DSCAS.

6a-ii) Describe whether and how "use" (including intensity of use/dosage) was defined/measured/monitored

"Usage will be examined for the myCompass group with respect to three indices, namely, frequency of logins, frequency of self-monitoring, and number of modules attempted."

6a-iii) Describe whether, how, and when qualitative feedback from participants was obtained

Qualitative data will be obtained using open ended questions asking about 'most' and 'least' liked features at post-intervention.

6b) CONSORT: Any changes to trial outcomes after the trial commenced, with reasons

The submitted manuscript describes the protocol for a randomised controlled trial. Hence, this item is not relevant at this stage.

7a) CONSORT: How sample size was determined

7a-i) Describe whether and how expected attrition was taken into account when calculating the sample size

"The RCT of myCompass yielded an average between-group effect size on symptom outcomes of d=0.5 [25]. Van Bastellar et al [22] also reported controlled effect sizes of an online intervention for people with diabetes in the vicinity of d=0.5 for diabetes-related distress and depressive symptoms. Assuming an attrition rate of 50% [48, 49], a total sample of 240 participants at follow-up (120 per arm) is the minimum required to detect between-group differences on self-report outcomes of .5 standard deviations with 80% power."

7b) CONSORT: When applicable, explanation of any interim analyses and stopping guidelines

The submitted manuscript describes the protocol for a randomised controlled trial. Hence, this item is not relevant at this stage.

8a) CONSORT: Method used to generate the random allocation sequence

"Randomisation to either myCompass or the active placebo intervention will be carried out after baseline measurement, according to a sequence generated by a computerized random-number generator [31] using permutated blocks of 2, 4, and 8. The randomisation process will be facilitated by a researcher not involved with the study. Participants will receive login details for their respective interventions by email."

8b) CONSORT: Type of randomisation; details of any restriction (such as blocking and block size)

"Randomisation to either myCompass or the active placebo intervention will be carried out after baseline measurement, according to a sequence generated by a computerized random-number generator [31] using permutated blocks of 2, 4, and 8. The randomisation process will be facilitated by a researcher not involved with the study. Participants will receive login details for their respective interventions by email."

9) CONSORT: Mechanism used to implement the random allocation sequence (such as sequentially numbered containers), describing any steps taken to conceal the sequence until interventions were assigned

"Randomisation to either myCompass or the active placebo intervention will be carried out after baseline measurement, according to a sequence generated by a computerized random-number generator [31] using permutated blocks of 2, 4, and 8. The randomisation process will be facilitated by a researcher not involved with the study. Participants will receive login details for their respective interventions by email."

10) CONSORT: Who generated the random allocation sequence, who enrolled participants, and who assigned participants to interventions

"Participants will receive login details for their respective interventions by email."

11a) CONSORT: Blinding - If done, who was blinded after assignment to interventions (for example, participants, care providers, those assessing outcomes) and how

11a-i) Specify who was blinded, and who wasn't

The study described will not be blinded for ethical reasons.

11a-ii) Discuss e.g., whether participants knew which intervention was the "intervention of interest" and which one was the "comparator"

Participants will be informed that they will receive one of two interventions, either focusing on psychological wellbeing or on healthy lifestyle. They will be informed that these interventions are being compared to see which one is better.

11b) CONSORT: If relevant, description of the similarity of interventions

"Developed by the research team to match myCompass on duration and mode of delivery, the program also contains practice activities, home tasks and factual SMS messages (sent to participants once-weekly) and a symptom check at four weeks, to replicate the interactivity of myCompass, but has no therapeutic content."

12a) CONSORT: Statistical methods used to compare groups for primary and secondary outcomes

"Analyses will be completed with SPSS 22 software. Chi-squares (categorical variables) and t-tests (continuous variables) will be used to compare demographic and disease-related variables and baseline scores on the outcome measures for the intervention and attention control groups.   Similar analyses will be performed comparing participants who do ('non-dropouts') and do not ('dropouts) return completed questionnaires at each of the post-intervention and follow-up assessments to explore possible biases in study attrition.

Outcomes at each time-point will be analysed on an intention-to-treat basis using linear mixed modelling (LMM; [50]), with timepoints as a within-group factor and intervention as a between-group factor.). In LMM, incomplete cases are included in the analysis, and all available data is used to obtain parameter estimates.   The interaction of time and study condition will be of primary interest in each analysis, with a significant interaction indicating a group difference in the pattern of change over time in the outcome of interest. Significant interactions will be explored using sets of Bonferroni adjusted comparisons of the two groups at post-intervention and 3-month follow-up. All effects will be tested at p < .05, with adjustment according to the number of contrasts in each set. Within- and between-group effect sizes will be calculate using Cohen's d (based on the pooled standard deviation)."

12a-i) Imputation techniques to deal with attrition / missing values

"Outcomes at each time-point will be analysed on an intention-to-treat basis using linear mixed modelling (LMM; [50]), with timepoints as a within-group factor and intervention as a between-group factor.). In LMM, incomplete cases are included in the analysis, and all available data is used to obtain parameter estimates."

12b) CONSORT: Methods for additional analyses, such as subgroup analyses and adjusted analyses

"Outcomes at each time-point will be analysed on an intention-to-treat basis using linear mixed modelling (LMM; [50]), with timepoints as a within-group factor and intervention as a between-group factor.). In LMM, incomplete cases are included in the analysis, and all available data is used to obtain parameter estimates."

RESULTS

13a) CONSORT: For each group, the numbers of participants who were randomly assigned, received intended treatment, and were analysed for the primary outcome

The submitted manuscript describes the protocol for a randomised controlled trial. Hence, this item is not relevant at this stage.

13b) CONSORT: For each group, losses and exclusions after randomisation, together with reasons

The submitted manuscript describes the protocol for a randomised controlled trial. Hence, this item is not relevant at this stage.

13b-i) Attrition diagram

The submitted manuscript describes the protocol for a randomised controlled trial. Hence, this item is not relevant at this stage.

14a) CONSORT: Dates defining the periods of recruitment and follow-up

The submitted manuscript describes the protocol for a randomised controlled trial. Hence, this item is not relevant at this stage.

14a-i) Indicate if critical "secular events" fell into the study period

The submitted manuscript describes the protocol for a randomised controlled trial. Hence, this item is not relevant at this stage.

14b) CONSORT: Why the trial ended or was stopped (early)

The submitted manuscript describes the protocol for a randomised controlled trial. Hence, this item is not relevant at this stage.

15) CONSORT: A table showing baseline demographic and clinical characteristics for each group

The submitted manuscript describes the protocol for a randomised controlled trial. Hence, this item is not relevant at this stage.

15-i) Report demographics associated with digital divide issues

The submitted manuscript describes the protocol for a randomised controlled trial. Hence, this item is not relevant at this stage.

16a) CONSORT: For each group, number of participants (denominator) included in each analysis and whether the analysis was by original assigned groups

16-i) Report multiple "denominators" and provide definitions

The submitted manuscript describes the protocol for a randomised controlled trial. Hence, this item is not relevant at this stage.

16-ii) Primary analysis should be intent-to-treat

"Group differences will be analysed on an intention-to-treat basis using mixed models repeated measures."

17a) CONSORT: For each primary and secondary outcome, results for each group, and the estimated effect size and its precision (such as 95% confidence interval)

The submitted manuscript describes the protocol for a randomised controlled trial. Hence, this item is not relevant at this stage.

17a-i) Presentation of process outcomes such as metrics of use and intensity of use

The submitted manuscript describes the protocol for a randomised controlled trial. Hence, this item is not relevant at this stage.

17b) CONSORT: For binary outcomes, presentation of both absolute and relative effect sizes is recommended

The submitted manuscript describes the protocol for a randomised controlled trial. Hence, this item is not relevant at this stage.

18) CONSORT: Results of any other analyses performed, including subgroup analyses and adjusted analyses, distinguishing pre-specified from exploratory

The submitted manuscript describes the protocol for a randomised controlled trial. Hence, this item is not relevant at this stage.

18-i) Subgroup analysis of comparing only users

The submitted manuscript describes the protocol for a randomised controlled trial. Hence, this item is not relevant at this stage.

19) CONSORT: All important harms or unintended effects in each group

The submitted manuscript describes the protocol for a randomised controlled trial. Hence, this item is not relevant at this stage.

19-i) Include privacy breaches, technical problems

The submitted manuscript describes the protocol for a randomised controlled trial. Hence, this item is not relevant at this stage.

19-ii) Include qualitative feedback from participants or observations from staff/researchers

The submitted manuscript describes the protocol for a randomised controlled trial. Hence, this item is not relevant at this stage.

DISCUSSION

20) CONSORT: Trial limitations, addressing sources of potential bias, imprecision, multiplicity of analyses

20-i) Typical limitations in ehealth trials

The submitted manuscript describes the protocol for a randomised controlled trial. Hence, this item is not relevant at this stage.

21) CONSORT: Generalisability (external validity, applicability) of the trial findings

21-i) Generalizability to other populations

The submitted manuscript describes the protocol for a randomised controlled trial. Hence, this item is not relevant at this stage.

21-ii) Discuss if there were elements in the RCT that would be different in a routine application setting

The submitted manuscript describes the protocol for a randomised controlled trial. Hence, this item is not relevant at this stage.

22) CONSORT: Interpretation consistent with results, balancing benefits and harms, and considering other relevant evidence

22-i) Restate study questions and summarize the answers suggested by the data, starting with primary outcomes and process outcomes (use)

The submitted manuscript describes the protocol for a randomised controlled trial. Hence, this item is not relevant at this stage.

22-ii) Highlight unanswered new questions, suggest future research

The submitted manuscript describes the protocol for a randomised controlled trial. Hence, this item is not relevant at this stage.

Other information

23) CONSORT: Registration number and name of trial registry

"Australian New Zealand Clinical Trials Registry ACTRN12614000974606; https://www.anzctr.org.au/Trial/Registration/TrialReview.aspx?id=366607"

24) CONSORT: Where the full trial protocol can be accessed, if available

"Australian New Zealand Clinical Trials Registry ACTRN12614000974606; https://www.anzctr.org.au/Trial/Registration/TrialReview.aspx?id=366607"

25) CONSORT: Sources of funding and other support (such as supply of drugs), role of funders

"This research was funded by an Australian Rotary Health Mental Health Research Grant."

X26-i) Comment on ethics committee approval

"The study protocol has been approved by the Ethics Committee at St Vincent's Hospital, which is certified by the National Health and Medical Research Council in Australia (HREC/14/SVH/31), and research governance bodies at each of the participating hospitals. The protocol is registered with the Australia and New Zealand Clinical Trials Registry (ACTRN12614000974606)."

x26-ii) Outline informed consent procedures

"Clinical staff at each service will provide potential participants with written information about the study and an invitation to take part during a routine visit. Information will describe the study and instruct interested individuals to access a study-specific website to complete an online consent form and screening survey."

X26-iii) Safety and security procedures

"If any participant indicates a significant worsening of their psychological distress (defined as a score > 19 on the PHQ-9 either midway through the intervention period, at post-intervention or follow-up) they will be sent an email from the research team advising them to contact their general practitioner (GP) to arrange face-to-face support. A second email (sent 3 days later) will seek confirmation that contact with the GP has been made. If no (or if no reply email is received), participants will be informed by email that the Principal Investigator will contact their nominated GP to recommend they receive appropriate face-to-face support."

X27-i) State the relation of the study team towards the system being evaluated

"None declared."
